# 

**Published:** 1946

**Authors:** 


| | Page |
|---|---|
| The Thirty-fourth Long Fox Memorial Lecture: Plant Hormones. By M. Skene, D.Sc. | 125 |
| "Night-Nurse's Paralysis": A Temporary Tonic Motor Paralysis. By G. de M. Rudolf, M.R.C.P., D.P.M., D.P.H. | 132 |
| Campaigning Notes, 1940–45. By Colonel Martin Herford, D.S.O., M.B.E., M.C., R.A.M.C. | 136 |
| Reviews of Books | 148 |
| Editorial Note | 150 |
| Obituary: Charles Edward Kynaston Herapath, M.C., M.D. (Lond.) | 152 |
| Meetings of Societies | 153 |
| The Medical Library of the University of Bristol | 157 |
| Publications Received | 160 |
| Local Medical Notes | 160 |

